# Q*uadruple* wild-type (WT) GIST: defining the subset of GIST that lacks abnormalities of KIT, PDGFRA, SDH, or RAS signaling pathways

**DOI:** 10.1002/cam4.325

**Published:** 2014-08-28

**Authors:** Maria A Pantaleo, Margherita Nannini, Christopher L Corless, Michael C Heinrich

**Affiliations:** 1 Department of Specialized, Experimental and Diagnostic Medicine, Sant'Orsola-Malpighi Hospital, University of BolognaBologna, Italy; 2 “Giorgio Prodi” Cancer Research Center, University of BolognaBologna, Italy; 3 Knight Cancer Institute, Oregon Health and Science University PortlandPortland, Oregon; 4 Division of Hematology and Oncology, Oregon Health and Science University PortlandPortland, Oregon

**Keywords:** BRAF, gastrointestinal stromal tumors, GIST, NF-1, quadruple negative, quadruple WT, RAS, SDH deficiency, SDHA, SDHB, wild type

## Abstract

A subset of GISTs lack mutations in the KIT/PDGFRA or RAS pathways and yet retain an intact succinate dehydrogensase (SDH) complex. We propose that these KIT/PDGFRA/SDH/RAS-P WT GIST tumors be designated as quadruple wild-type (WT) GIST. Further molecular and clinicophatological characterization of quadruple WT GIST will help to determine their prognosis as well as assist in the optimization of medical management, including clinical test of novel therapies.

## Introduction

Approximately, 85–90% of gastrointestinal stromal tumors (GISTs) in adults harbor mutant KIT or platelet-derived growth factor receptor alpha (PDGFRA) oncoproteins 1. The remaining adult cases and the vast majority of pediatric GISTs do not harbor mutations in these receptors and are often referred to as KIT/PDGFRA wild-type (WT) GISTs 1,2. Amongst the WT GISTs, at least two other different subgroups with well-defined molecular hallmarks have been described. Approximately, 15% of these cases harbor an activating mutation in BRAF, or more rarely, a RAS gene 3. In addition, WT GIST can arise in the context of the syndromic neurofibromatosis type I (NF1) disease, associated with loss of function of the NF1 protein due to genomic inactivation of both NF1 alleles 4. Collectively, GISTs with mutations in BRAF/RAS or NF1 can be referred to as the RAS-pathway (RAS-P) mutant GIST.

Between 20% and 40% of KIT/PDGFRA WT GISTs show loss of function of the succinate dehydrogenase complex (SDH), manifested by the loss of subunit B (SDHB) protein expression. These tumors are designated as *SDH-deficient* GISTs or SDHB-negative GISTs based on their immunohistochemical (IHC) status. Some investigators have designated SDHB IHC-negative GISTs as type 2 GISTs. Furthermore, SDH-deficient GISTs have distinctive clinicopathological features characteristic pathological, and clinical characteristics, including a predilection for young women, gastric localization, mixed epithelioid and spindle cell morphology, diffuse KIT and ANO1 (DOG1) IHC positivity, frequent lymph node metastases, and an indolent course of disease even when metastases are present 5,6. Moreover, the SDHB IHC-negative GIST is characterized by over expression of the insulin growth factor 1 receptor (IGF1R) 7,8. The most frequent identifiable molecular events found in SDHB-deficient GISTs are germline and/or somatic loss-of-function mutations in any of the four SDH subunits. (A, B, C, or D) 9,10. Recently, other molecular events associated with SDH deficiency have been reported, including genome-wide DNA hypermethylation and a specific microRNA profile 11,12.

Tumors with SDHA mutations comprise the most common subtype of SDH-deficient GIST, and demonstrate loss of SDHA protein expression in addition to the loss of SDHB protein expression 13,14. The SDHB IHC-negative/SDHA IHC-positive subgroup is histologically similar to SDHA IHC-negative GIST, but with a lesser female prevalence. Many of these GISTs arise in the context of the Carney–Stratakis Syndrome (the dyad of GIST and paraganglioma), and are characterized by germline SDHB, SDHC or SDHD-inactivating mutations. They also occur in the context of the Carney Triad (gastric GIST, paraganglioma, and pulmonary chondroma), which do not harbor SDHx-mutations 15,16. Recently, Haller et al. have reported hypermethylation of SDHC as a novel mechanism of tumor development in Carney Triad 17.

KIT/PDGFRA WT GISTs lacking abnormalities of the SDH complex are SDHB IHC-positive and have been referred to as *type 1* GIST. This SDHB IHC-positive subgroup includes NF1-mutated GIST, which commonly present in the small bowel in a multifocal manner and are negative for IGF1R staining. This subgroup also includes sporadic KIT/PDGFRA WT GIST arising anywhere in the gastrointestinal tract in adult patients 6.

Based on the above array of molecular markers, it has become apparent that approximately 5% of all GISTs lack mutations in the KIT exons 8, 9, 11, 13, 14, 17/PDGFRA exons 12, 14, 18 or RAS pathways (BRAF exons 11, 15/RAS exons 2, 3 or NF1), and yet retain an intact SDH complex (SDHB IHC positive, no mutations of SDHA/B/C/D).

**Figure 1:**
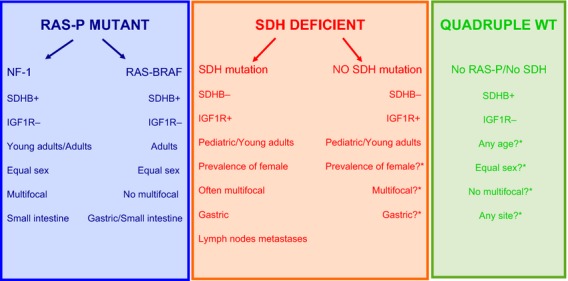
Current characterization of KIT/PDGFRA WT GIST. *More data should be accumulated.

We propose that these KIT/PDGFRA/SDH/RAS-P WT GIST tumors be designated as *quadruple WT* GIST, or *quadruple negative* GIST, in contrast to other categories of GISTs characterized by oncogenic abnormalities of at least of one of the four pathways (KIT mutant, PDGFRA mutant, SDH deficient, or RAS/BRAF/NF1 mutant GISTs) (Fig.1). The pathogenesis and underlying biology of quadruple WT GISTs is currently unknown. Moreover, descriptive clinical and pathological data for this group have not been defined. However, the absence of molecular events in the four known pathways suggests that this entity represents a completely different type of GIST. Genome wide studies of gene expression, copy number variation, and/or transcriptome sequencing may be useful to better characterize quadruple WT GISTs and to identify their underlying molecular abnormalities. Given their rarity, clinical and pathological data should be analyzed from large series of quadruple WT GIST to help identify their specific clinicopathological features. Discovering the novel molecular alterations that characterize quadruple WT GIST will help to define their clinical behavior/prognosis as well as to aid in the evaluation of conventional as well as novel GIST medical treatments. As with other subgroups of GIST, we propose that further specific studies of quadruple WT GIST will help to optimize diagnosis and medical management.

## Conflict of Interest

None declared.
